# Intracellular fate of carbon nanotubes inside murine macrophages: pH-dependent detachment of iron catalyst nanoparticles

**DOI:** 10.1186/1743-8977-10-24

**Published:** 2013-06-25

**Authors:** Cyrill Bussy, Erwan Paineau, Julien Cambedouzou, Nathalie Brun, Claudie Mory, Barbara Fayard, Murielle Salomé, Mathieu Pinault, Mickaël Huard, Esther Belade, Lucie Armand, Jorge Boczkowski, Pascale Launois, Sophie Lanone

**Affiliations:** 1 Inserm U955 Équipe 4, Faculté de Médecine, 8 rue du Général Sarrail, 94010 Créteil, France; 2 UMR S955, Faculté de Médecine, Université Paris-Est, Créteil F-94000, France; 3 Laboratoire de Physique des Solides, UMR CNRS 8502, Université Paris-Sud 11, F-91405 Orsay cedex, France; 4 X-ray Imaging Group, European Synchrotron Radiation Facility, BP 220, F-38043 Grenoble Cedex, France; 5 CEA, IRAMIS, SPAM, Laboratoire Francis Perrin (CEA-CNRS URA 2453), 91191 Gif-sur-Yvette, France; 6 Service de Physiologie Explorations Fonctionnelles, AP-HP, Hôpital Henri Mondor, F-94010 Créteil, France; 7 Service de pneumologie et pathologie professionnelle, Hôpital Intercommunal de Créteil, Créteil F-94000, France; 8 Nanomedicine laboratory, Centre for Drug Delivery Research, UCL School of Pharmacy, University College London, London WC1N 1AX, UK; 9 UMR 5257 CEA/CNRS/UMII/ENSCM, Centre de Marcoule, Institut de Chimie Séparative de Marcoule, BP 17171, F-30207 Bagnols sur Cèze Cedex, France

**Keywords:** Carbon nanotubes, Degradation, Iron catalyst nanoparticles, Toxicity, Lysosome acidification

## Abstract

**Background:**

Carbon nanotubes (CNT) are a family of materials featuring a large range of length, diameter, numbers of walls and, quite often metallic impurities coming from the catalyst used for their synthesis. They exhibit unique physical properties, which have already led to an extensive development of CNT for numerous applications. Because of this development and the resulting potential increase of human exposure, an important body of literature has been published with the aim to evaluate the health impact of CNT. However, despite evidences of uptake and long-term persistence of CNT within macrophages and the central role of those cells in the CNT-induced pulmonary inflammatory response, a limited amount of data is available so far on the CNT fate inside macrophages. Therefore, the overall aim of our study was to investigate the fate of pristine single walled CNT (SWCNT) after their internalization by macrophages.

**Methods:**

To achieve our aim, we used a broad range of techniques that aimed at getting a comprehensive characterization of the SWCNT and their catalyst residues before and after exposure of murine macrophages: X-ray diffraction (XRD), High Resolution (HR) Transmission Electron Microscopy (TEM), High Angle Annular Dark Field-Scanning TEM (HAADF-STEM) coupled to Electron Energy Loss Spectroscopy (EELS), as well as micro-X-ray fluorescence mapping (μXRF), using synchrotron radiation.

**Results:**

We showed 1) the rapid detachment of part of the iron nanoparticles initially attached to SWCNT which appeared as free iron nanoparticles in the cytoplasm and nucleus of CNT-exposed murine macrophages, and 2) that blockade of intracellular lysosomal acidification prevented iron nanoparticles detachment from CNT bundles and protected cells from CNT downstream toxicity.

**Conclusions:**

The present results, while obtained with pristine SWCNT, could likely be extended to other catalyst-containing nanomaterials and surely open new ways in the interpretation and understanding of CNT toxicity.

## Background

Carbon nanotubes (CNT) are a family of nanomaterials featuring a large range of length, diameter, numbers of walls (single-walled -SWCNT-, double-walled -DWCNT- or multi-walled -MWCNT-) and, most often metallic impurities coming from the catalyst used for their synthesis. They exhibit unique physical properties, which have already led to their extensive use in composite materials [1]. Moreover, prospective applications are also expected in electronics, for flat-panel displays, energy storage, water pollutants adsorbents, as well as in the nanomedicine field where CNT could be used for drug and non-viral gene delivery or for diagnosis [2]. Due to the expansion of CNT usage and the resulting increase of potential human exposure, numerous investigations have, in recent years, aimed at evaluating the health impact of CNT in an occupational, environmental or biomedical context.

*In vivo* studies, essentially performed with animals exposed *via* the respiratory route have demonstrated that pulmonary exposure to CNT led to an immediate macrophage-mediated inflammatory response characterized mainly by a prominent macrophage influx in the bronchoalveolar fluid, together with an internalization of the CNT by the resident or attracted macrophages [3-8]. At later stages, respiratory exposure to CNT can lead to the development of pulmonary fibrosis and/or granuloma [9-13]. The presence of CNT in macrophages at the site of exposure or distributed throughout the body has been documented up to 24 months after the initial exposure [12]. However, despite evidences of uptake and long-term persistence of CNT within macrophages, together with the central role of those cells in the CNT-induced inflammatory response, a limited amount of data is available so far on the fate of CNT inside macrophages. Whether internalized CNT are biopersistent or could be biotransformed have been indeed poorly investigated in macrophages. This is a critical and major gap in the actual knowledge because by-products resulting from the potential CNT transformation inside cells could have biological effects *per se* and thus be essential determinants in the toxicological profile of CNT.

The overall aim of our study was therefore to investigate the fate of SWCNT after their internalization by macrophages. We hypothesized that the residence of CNT inside cellular acidic compartments will lead to physicochemical and structural modifications of the internalized CNT. This was evaluated by using a broad range of techniques that aimed at getting a comprehensive characterization of the pristine SWCNT used, and of their metallic catalyst residues before and after exposure of murine macrophages. Our results show that 1) part of the iron-based catalyst nanoparticles initially attached to SWCNT rapidly appeared as free iron-based nanoparticles in the cytoplasm and nucleus of CNT-exposed murine macrophages, and that 2) the blockade of intracellular acidification processes prevented iron nanoparticles detachment from the CNT and also protected the cells from some potentially deleterious downstream effects induced by CNT. These data provide the first evidence of a biological processing of pristine CNT inside macrophages *via* a pH-dependent mechanism. Moreover, they also demonstrate the determinant role of by-products of this bio-processing in CNT toxicological profile.

## Results and discussion

**Figure 1:**
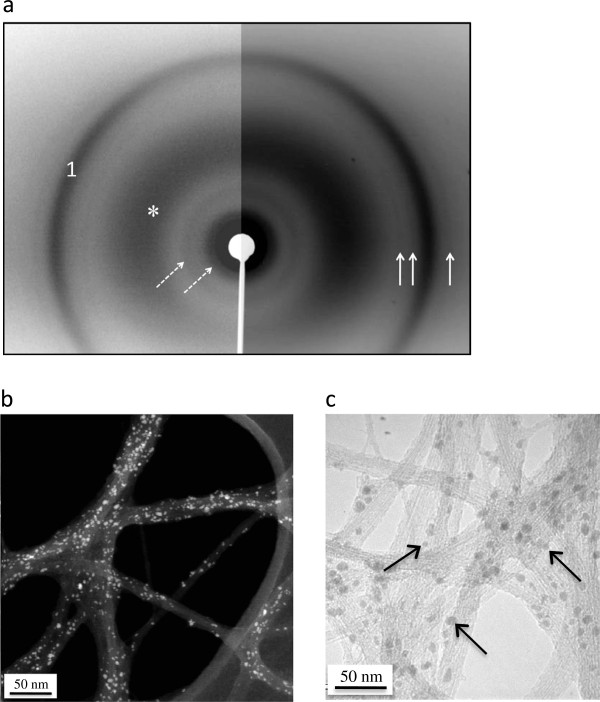
**X-ray diffraction and electron microscopy of as-produced pristine SWCNT.** Panel **a**: X-ray diffraction pattern of a SWCNT powder placed in a glass capillary. The image is made of two patterns recorded for different durations, allowing the visualization of intense peaks (left part of the image) as well as much weaker ones (right). The two dotted arrows point towards diffraction peaks, around 0.5 and 0.9 Å^-1^, from the CNT bundles, the large bump (star) comes from the glass capillary and peak 1, at wave-vector Q ~ 3 Å^-1^, can be attributed to both CNT and iron-based nanoparticles. The three plain arrows point toward diffraction peaks characteristic of cementite Fe_3_C (see e.g. [16]). The widths of the peaks are resolution-limited and they correspond to particle sizes of about 5 nm and above: one mainly probes here the bigger particles (Φ ≥ 5 nm). Panel **b**: HAADF-STEM image showing tiny white spots corresponding to iron-based nanoparticles covering lightly contrasted bundles of SWCNT deposited on a TEM grid coated with a lacey carbon film. Nanoparticle diameters range from 1 to 10 nm. Panel **c**: HRTEM image of bundles of SWCNT. Arrows show iron-based catalyst nanoparticles on the sidewalls of CNT.

The diameter of the pristine SWCNT used in the present study has been previously evaluated by some of us [14] and estimated to be in the 0.8-1.2 nm range, while their individual length was in the 100–1000 nm range, according to supplier information. Thermogravimetric analysis measured an iron content of about 25-wt%, confirming the unpurified nature of the sample. X-ray diffraction (XRD) experiments revealed intense peaks at low wave-vector Q values characteristic of SWCNT organized in bundles (for a more detailed analysis, see [15]), while weaker peaks at higher wave-vectors corresponded to the signal of iron-based nanocrystals of cementite Fe_3_C (Figure 1a). No Bragg peak was observed at 1.8 Å^-1^, i.e. at wave-vectors corresponding to the stacking distance between the graphenic planes constitutive of graphite. This indicates that samples are free of graphitic impurities. Numerous iron-based particles (diameter in the 1–10 nm range), originating from the decomposition of iron pentacarbonyl during the synthesis, were present in the form of nanoparticles on the sidewalls of CNT, as observed in High Angle Annular Dark Field-Scanning Transmission Electron Microscopy (HAADF-STEM) and High Resolution Transmission Electron Microscopy (HRTEM) images (Figure 1b and 1c respectively). These residual iron nanoparticles were attached to CNT bundles through one or several carbon shells, as previously shown in ref. [14]. The iron chemical nature of these nanoparticles was confirmed by Electron Energy Loss Spectroscopy (EELS), which exhibits a doublet of peaks corresponding to transitions from the Fe 2p core levels to the Fe 3d levels (Additional file 1: Figure S1).

**Figure 2:**
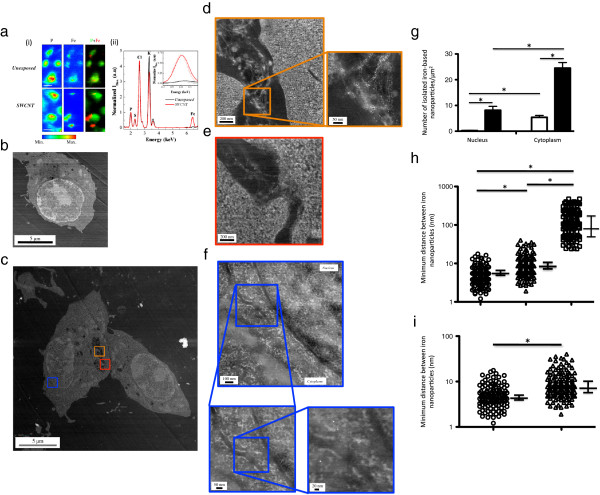
**X-ray fluorescence analysis, and HAADF-STEM images of unexposed and SWCNT-exposed cells.** Panel **a**-**i**: typical μXRF maps of P and Fe of macrophages unexposed (upper line) or exposed (lower line) to 50 μg/ml SWCNT for 24 hours (the color scale is a temperature scale, ranging from blue for low concentrations in the element of interest to red for high concentrations); right: P (green) and Fe (red) correlation images. The size of a pixel is 0.25 μm^2^ (scale bar: 10 μm). Panel **a**-**ii**: X-ray fluorescence spectra integrated exclusively over the scanned cells, normalized to the phosphorus signal. Positions of Kα fluorescence peaks of phosphorus (P), sulfur (S), chlorine (Cl), potassium (K) and iron (Fe) are indicated. The inset represents zoomed areas around the positions of the Kα fluorescence peak of iron together with fits of its contribution (dashed lines). Panel **b** to **f**: representative HAADF-STEM images of unexposed cell (Panel **b**), and low (Panel **c**) or high (Panels **d**, **e** and **f**) magnification of cells exposed to SWCNT for 24 hours. In Panel **f**, nuclear membrane is clearly visible, separating cytoplasm (left part of the image) from nucleus (right part of the image). Panel **g**: quantification of isolated iron nanoparticles in the nucleus and the cytoplasm of cells exposed to SWCNT for 3 hours (open bars) or 24 hours (dark bars). Panel **h**: quantification of minimum distance between iron nanoparticles observed with SWCNT alone (open circles), SWCNT in vesicles (open triangles) or with nanoparticles in cytoplasm or nucleus (open squares). *: p < 0.0001 between groups. Panel **i**: higher magnification of Panel **h** for SWCNT alone and SWCNT in cell vesicles. *: p < 0.001 between groups.

To assess at the scale of several cells if SWCNT were present in SWCNT-exposed cells, we took advantage of iron catalyst residues attached to SWCNT and performed synchrotron-based micro X-ray fluorescence (μXRF) experiments on macrophages exposed to 0 (unexposed) or 50 μg/mL of pristine SWCNT. Figure 2a-i shows typical μXRF maps obtained for phosphorus (P) and iron (Fe) elements in unexposed (upper panel) and SWCNT-exposed macrophages (lower panel) after 24 hours exposure. The cellular shape can be drawn by the P map since phosphorus represents a characteristic element of cellular components [17]. Moreover, endogenous iron was detectable and rather uniformly distributed in unexposed cells. For SWCNT-exposed cells, Fe maps presented both iron-rich zones co-localized with the P signal attributed to endogenous iron and specific iron-rich zones, close to the external border or outside the cells, which can be attributed to the CNT presence. Micro-XRF spectra integrated over regions where only cells were present (Figure 2a-ii) further confirmed the higher cellular content of iron associated with SWCNT exposure (red line), as compared to unexposed cells (black line). Similar results were obtained after 3 hours of exposure (data not shown).

To further evaluate the fate of SWCNT at the single cell and subcellular levels, we used HAADF-STEM to image cell sections after 3 or 24 hours of exposure. Figure 2b and c show typical HAADF-STEM images obtained with unexposed macrophages (Figure 2b and Additional file 2: Figure S2a) or with macrophages exposed to SWCNT over a 24 hours period (Figure 2c). Similar images were obtained after a 3 hours exposure (data not shown). Higher magnifications of cellular vesicles present in Figure 2c (orange and red squares, Figure 2d and e) clearly showed the presence of SWCNT bundles within the vesicles (inset Figure 2d), with small round nanoparticles (tiny white dots) attached to them, all along the CNT length. The chemical nature of these nanoparticles was checked using EELS measurements and displayed the characteristic Fe 2p to Fe 3d peak doublet of iron at 710 and 725 eV, similar to that found in the original material (Figure S1 and data not shown). Moreover, a typical image obtained in cytoplasmic and nuclear regions of cells exposed to SWCNT (zone containing cytoplasm and nucleus, as delimited by the blue square in Figure 2c) is shown in Figure 2f (and Additional file 2: Figure S2). Interestingly, it revealed isolated iron nanoparticles in both compartments; the chemical nature of the nanoparticles (*i*.*e*. iron) was here also confirmed by EELS analysis. In addition, the apparently free nanoparticles were similar in size to those initially attached to pristine CNT (1–10 nm, Figure 1b). It is noteworthy that we did not observe (HAADF-STEM and HRTEM analysis) any detachment of iron catalyst particles following CNT sample preparation (before cell exposure; gentle bath sonication treatment), which led us to conclude that detachment happened during (and probably because of) interaction with cells. To the best of our knowledge, such cellular detachment of metal based catalyst nanoparticles from CNT backbone has never been described in cells.

Apart from within vesicles, the intracellular localization of iron nanoparticles was mainly cytoplasmic, but a significant amount of these nanoparticles was also observed inside the nucleus after 24 hours. The quantification of the isolated nanoparticles showed that their total number significantly increased with time (from 3 to 24 hours), demonstrating a time-dependent process (Figure 2g). Interestingly, in cytoplasm and nucleus, iron nanoparticles did not appear to follow anymore the fibrillar shape of nanotube bundles as observed in vesicles (Figure 2d), but were rather isolated. Therefore, we further quantified the minimal distance between two nanoparticles in SWCNT before exposure, in SWCNT within vesicles, and in cytoplasm and nucleus (using ImageJ analysis of HAADF-STEM images; Figure 2h and 2i). As compared to what was observed with SWCNT (original material before cell exposure) or with SWCNT inside vesicles, the mean minimal distance between iron nanoparticles observed in the cytoplasm and/or nucleus was clearly higher (Figure 2h). In addition, the mean minimal distance was also slightly but significantly higher for iron nanoparticles observed in SWCNT-containing vesicles than for nanoparticles attached to SWCNT (Figure 2i). These results suggested that following the cellular uptake of CNT, the iron-based particles start to detach from the CNT backbone within the vesicles, and then translocate to the cytoplasm and eventually into the nucleus.

**Figure 3:**
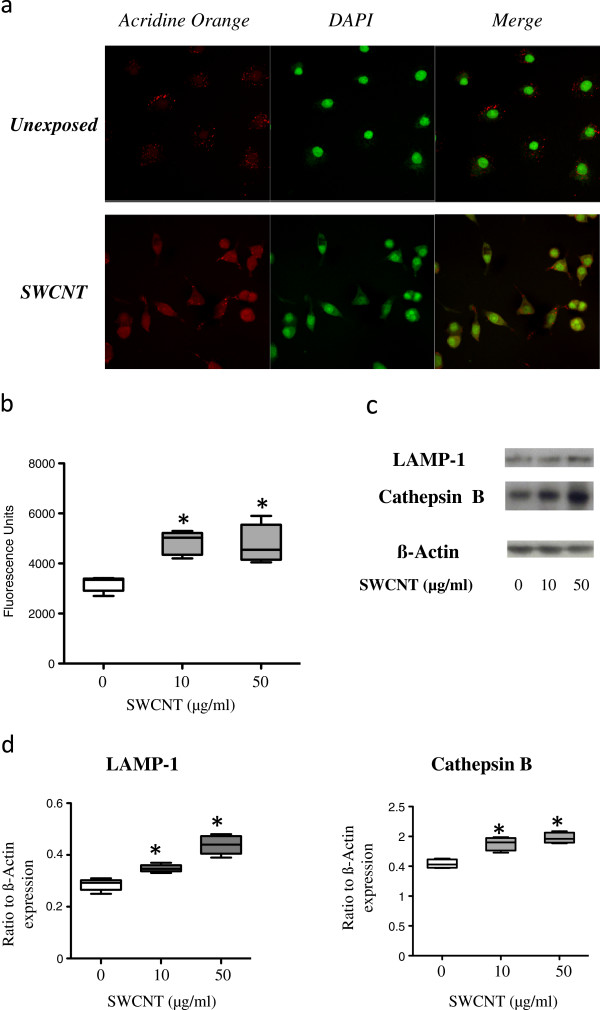
**Evaluation of lysosomal activation in SWCNT-exposed cells.** Panel **a**: Epi-fluorescence images of macrophages unexposed (upper line) or exposed (lower line) to 50 μg/ml SWCNT for 24 hours stained with Acridine Orange (for lysosomes, in red) and DAPI (RNA/DNA, in green). Merge: combination of Acridine Orange and DAPI staining (overlapping appeared yellow). Panel **b**: Cathepsin activity in macrophages exposed to SWCNT for 3 hours. *: p < 0.001 between groups. Panel **c**: Western Blot images of LAMP-1 (120 kDa) and Cathepsin B (37 kDa) expression in macrophages exposed for 24 hours to SWCNT. ß-Actin is given as internal standard. Panel **d**: quantification of LAMP-1 and Cathepsin B expression, normalized to ß-Actin expression. *: p < 0.05 between groups.

Because acidic treatment is a process commonly used to remove metallic impurities from as-produced pristine CNT samples [14,18], we next hypothesized that the acidic environment present in phagolysosomes (pH 4.5) could be responsible for the detachment of the iron-based nanoparticles from the pristine CNT following their internalization. To test our hypothesis, we first assessed whether acidic phagolysosomes appeared in macrophages exposed to SWCNT. After 3 and 24 hours exposure to CNT, epi-fluorescence images displayed a concentration and the reorganization of acridine orange molecules coherent with an acidification of the lysosomes [19] (Figure 3a, and data not shown). The lysosomal activation was also confirmed by the dose-dependent significant increase in Cathepsin activity [19], observed in CNT-exposed macrophages (Figure 3b, p < 0.001 versus unexposed cells). Finally, the formation of phagolysosomes was further illustrated by the increased expression of two lysosomal proteins [20]: Cathepsin B and Lysosome-Associated Membrane glycoprotein 1 (LAMP-1, Figure 3c and d, p < 0.05 exposed versus unexposed cells). Taken together, these results demonstrate the acidification of macrophage intracellular compartments after exposure to SWCNT.

**Figure 4:**
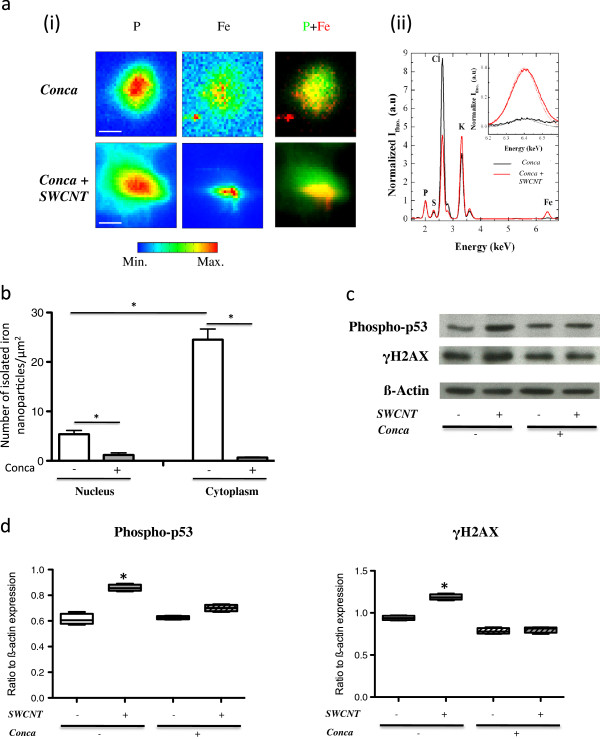
**Protective effect of Concanamycin A.** Panel **a**-**i**: typical μXRF maps of P and Fe of macrophages unexposed (upper line - Conca) or exposed (lower line - Conca + SWCNT) to 50 μg/ml SWCNT for 3 hours in presence of 10 nM Concanamycin A (the color scale is a temperature scale, ranging from blue for low concentrations in the element of interest to red for high concentrations); right: P (green) and Fe (red) correlation images. The size of a pixel is 0.25 μm^2^ (scale bar: 10 μm). Panel **a**-**ii**: X-ray fluorescence spectra integrated exclusively over the scanned cells. Positions of Kα fluorescence peaks of phosphorus (P), sulfur (S), chlorine (Cl), potassium (K) and iron (Fe) are indicated. The inset represents zoomed areas around the positions of the Kα fluorescence peak of iron together with fits of its contribution (dashed lines). Panel **b**: quantification of isolated iron nanoparticles in the nucleus and the cytoplasm of macrophages exposed to SWCNT for 3 hours in absence (open bars) or presence (grey bars) of 10 nM Concanamycin A. *: p < 0.001 between groups. Panel **c**: Western Blot images of Phospho-p53 (53 kDa) and γH2AX (17 kDa) expression in macrophages exposed for 3 hours to 50 μg/ml SWCNT, in presence (+) or absence (−) of 10 nM Concanamycin A. ß-Actin is given as internal standard. Panel **d**: quantification of Phospho-p53 and γH2AX expression in Western Blot, normalized to ß-Actin expression. *: p < 0.05 between groups. Conca: Concanamycin A.

To further address the role of acidification, we performed experiments in presence of Concanamycin A, a potent inhibitor of intracellular pH acidification [21]. As shown in Figure 4a, μXRF maps and associated fluorescence spectra obtained in presence of Concanamycin A were similar to those obtained in absence of this inhibitor (Figure 2a-i), showing that the blockade of intracellular acidification did not prevent CNT uptake by macrophages. Nevertheless, the quantification of isolated iron nanoparticles showed that their total number significantly decreased in Concanamycin A-treated cells as compared to Concanamycin A non-treated cells, for both the nucleus and cytoplasm compartments (Figure 4b, p < 0.001 for treated versus untreated cells). This suggested that the detachment of nanoparticles and their subsequent distribution outside vesicles were related to pH acidification. Moreover, Concanamycin A treatment during CNT exposure was accompanied by a protection against CNT-induced phosphorylation of p53 and H2AX proteins which are both biomarkers for initiation of DNA repair processes [22] (Figure 4c and 4d). Those findings first indicated that inhibition of pH acidification protects macrophages from nuclear damages associated with SWCNT exposure. Secondly, they suggested that these deleterious effects might be related to the release of isolated nanoparticles from CNT due to the acidic environment inside lysosomes. We then further investigated whether the effects on DNA could be mediated by iron-related oxidative stress-dependent mechanisms. Expression of Ferritin heavy chain (H-Ferritin), which catalyzes the first step in ionic iron storage [23], and that of Heme Oxygenase (HO)-1 were both unmodified in macrophage exposure to SWCNT, in presence or absence of Concanamycin A (Additional file 3: Figure S3), which ruled out the possibility of an oxidative stress-driven mechanism.

Taken together, our results emphasize that, despite their initial encapsulation in carbon shells, catalyst iron nanoparticles can be detached from the SWCNT backbone via a pH-dependent mechanism and participate actively in the CNT-associated toxicity. Our study thus demonstrates for the first time that pristine CNT can be biologically modified inside macrophages *via* a lysosomal-dependent mechanism. Such results are particularly relevant in the context of an occupational exposure to as produced CNT, but are also likely to be part of a more global ability of macrophages to degrade carbon nanomaterial products.

In the last few years, different groups have demonstrated the possible enzyme-catalyzed oxidative biodegradation of CNT, although essentially in abiotic conditions [24-29]. However, these different studies focused mainly on the degradability of the graphitic lattices of CNT; and no specific data have been reported on the fate of the metal catalyst residues that are initially attached to the pristine CNT, although these residues are highly suspected to be essential determinants of the toxicological profile of non-purified CNT [7]. Moreover, none of these studies have focused on the fate of CNT inside macrophages, despite the central role of macrophages in CNT-induced inflammatory response [3-8]. In the present study performed with pristine SWCNT, isolated iron-based nanoparticles were observed within exposed cells. Importantly, these isolated iron nanoparticles, although similar in size to those initially attached to pristine CNT, were not organized following CNT fiber shape. This individualization suggests that the iron nanoparticles were either not anymore linked to the CNT backbone, or that they were attached to a shorter CNT backbone that could result from a concomitant graphitic lattice oxidative biodegradation as previously described [24,28,30]. Although we cannot completely exclude a concomitant degradation of the backbone owing to the methodologies used in the present study, the first proposition is by far the most likely since the carbon shells around nanoparticles are much more defective than the backbone of pristine CNT themselves and will thus be submitted first to degradation processes [28]. Indeed, defects or functionalized sites in the graphitic lattice of CNT appear to be mandatory to initiate the enzyme-mediated oxidative biodegradation of CNT backbone [28]. Notably, enzyme-catalyzed biodegradation of pristine material was only possible after introduction of oxidative defects in the graphitic lattice [28]. Although further studies are needed to fully characterize the mechanism of detachment observed here, our data strongly suggest a role for intracellular acidification in the detachment of iron nanoparticles, since Concanamycin A treatment abolished almost completely this event. We can also speculate that such acidification may allow the activation of pH-sensitive enzymes that could, in turn, attack the CNT backbone, but this hypothesis would need further investigations.

The presence of free and bioavailable iron nanoparticles in different cell compartments could have important pathophysiological consequences. The presence of small iron nanoparticles in the nuclear compartment suggests the possible penetration through ion channels or diffusion through pores in the nuclear membrane [31]. Such event could have severe consequences in case of interaction with DNA [32], as suggested by the activation of p53 and H2AX pathways in our experimental settings, and could therefore explain the genotoxic effects often associated with CNT exposure [33-37]. Interestingly, iron oxide particles have been shown to dissolve inside murine alveolar macrophages [38], suggesting a potential additional mechanism of toxicity related to the interaction of soluble iron with proteins. However, since the intracellular levels of the iron storage protein H-Ferritin were not increased in SWCNT-exposed macrophages, the generation of ionic iron from the detached iron nanoparticles seems unlikely, at least within the timeframe of exposure used here. This was also further evidenced by the absence of HO-1 induction, suggesting the absence of an iron ion related oxidative stress-driven mechanism [39,40]. Consequently, our data strongly support the idea that iron-based nanoparticles, and not solubilized iron, are important determinants of the SWCNT-induced biological effects observed in the present study.

## Conclusion

In conclusion, our study provides the first evidence of biological transformation of SWCNT inside macrophages, as a result of an intracellular process governed by pH acidification, leading to catalyst nanoparticles detachment from CNT backbones. Our findings therefore demonstrate that although iron catalyst residues are initially isolated from biological media thanks to their surrounding carbon shells, they become more accessible to the biological environment owing to modifications following the uptake and residence of CNT bundles in acidic cell compartments. The present results, while obtained with pristine SWCNT, could likely be extended to other catalyst-containing nanomaterials and surely open new ways in the interpretation and understanding of CNT toxicity.

## Methods

### Carbon nanotubes

SWCNT were produced by the HiPCO process (HiPCO stands for ‘high pressure carbon monoxide’), which is a Chemical Vapor Deposition (CVD) process using continuous flowing carbon monoxide as the carbon feedstock and a small amount of iron pentacarbonyl as the iron-containing catalyst precursor at high pressure. Two batches of as-produced (raw grade) nanotubes were purchased from Carbon Nanotechnologies Incorporated (CNI® Buckytubes, Houston-TX, USA). Our high resolution transmission electron microscopy (HR-TEM) analysis (Akashi Topcon EM-002B microscope) has showed that iron is present in the form of nanoparticles on the side-walls of nanotube bundles [17], encapsulated in carbon shells [14]. Pristine SWCNT used in the present study were similar to those used in ref [17][14] and [15], but came from different batches.

### Carbon nanotube suspension

SWCNT were suspended at 250 μg/ml in serum-free cell culture medium (DMEM GlutaMAX^™^, Gibco LifeTechnologies). CNT suspensions were then rotated for 30 s, sonicated (RLI 275 sonication bath, LIREC, France) for 20 minutes under temperature-controlled conditions (+4°C), with 15 seconds interruption every 5 minutes for rotating steps. At the end of sonication, CNT suspensions were diluted at dedicated concentrations in serum-free cell culture medium, just before cell exposure. Scanning transmission electron microscopy (STEM) images (VG-HB501 microscope) were obtained on CNT before and after their gentle sonication and revealed no modification between the two samples.

### Cell culture conditions

Murine macrophages (RAW 264.7 cell line, LGC Promochem/ ATCC) were cultured for 3 or 24 hours in cell culture medium (DMEM GlutaMAX^™^, Gibco LifeTechnologies) supplemented with 100 U/ml penicillin, 100 μg/ml streptomycin and 10% heat-inactivated foetal bovine serum (PAA Laboratories GmbH) under standard cell culture conditions (humidified incubator at 37°C under 5% CO2). In a subset of experiments, cells were pretreated for 1 hour with 10 nM Concanamycin A (Sigma-Aldrich, France) before CNT exposure. In all experiments, the volume of culture medium was calculated to be 200 μl/cm^2^ of cell surface. (i.e. 5 ml for a 25 cm^2^ culture flask).

### X-ray diffraction

X-ray diffraction (XRD) experiments were performed using a rotating molybdenium anode generator (Rigaku). The MoKα wavelength (λ = 0.711 Å) was chosen to minimize iron fluorescence (as compared to CuKα wavelength). It is selected using a doubly-bent graphite monochromator, ensuring high flux. Scattering by air being non negligible compared to that by CNT, experiments were performed under vacuum to optimize the signal/background ratio. CNT powder was placed in a glass capillary and studied in transmission. The data were recorded on planar imaging plates.

### Thermogravimetric analysis

Thermogravimetric (TGA) analyses were performed under flowing air up to 1000°C (SETARAM apparatus) to determine the iron content in CNT powders by measuring the remaining weight corresponding to iron oxide after oxidative treatment.

### Electron microscopy and spectroscopy

For ultrastructural observations, cells were seeded at 250 000 cells/ml on 60 mm diameter cell culture Petri dishes (TPP, ATGC biotechnologie, France) and cultured for 24 hours in serum condition before exposure. After 3 or 24 hours exposure to 10 μg/ml serum-free CNT suspensions or to serum-free cell culture medium, macrophages monolayer were fixed with 2% glutaraldehyde / 2% paraformaldehyde in cacodilate buffer, dehydrated, embedded in epoxy Resin (Epon 812) and then sectioned using an ultramicrotome at 40–50 nm on 400-mesh copper TEM grids for high resolution imaging by High Angle Annular Dark Field-Scanning TEM (HAADF-STEM). HAADF-STEM images were performed on a VG-HB501 microscope equipped with a tungsten cold field-emission gun operated at 100 keV with a sub-nanometer incident probe (0.5-1 nm). Electron Energy Loss Spectroscopy (EELS) spectra were recorded with a modified Gatan spectrometer. Spectra were recorded so as to cover a large energy range, with an energy resolution of about 1 eV. To highlight the presence of nanoparticles for their quantification, images were further processed using the ‘differentials’ tool named directional gradient (angle degree 0.0°) in Gatan digital micrograph software. Pixel intensities in each STEM pictures are related to the atomic number of the material imaged. Using this software tool, nanoparticles with high atomic number were thus highlighted compared to the background (biological material or resin with low atomic numbers). Quantitative evaluation of the minimal distance measurable between two catalyst particles (2–4 nm each) was performed using ImageJ software (National Institutes of Health, USA), on STEM images with the highest magnifications (see Figure 2f as an example), where resolution allows the clear identification of nanoparticles (40 images analyzed for each time points, 2 independent investigators). Two main compartments were considered for distance calculation: vesicles (with clearly identifiable SWCNT) and the rest of the cell (cytoplasm and nucleus). Particles that were on the edges of these compartments were excluded. Data are represented as individual points and median ± quartile. Quantitative evaluation of the number of isolated particles was realized manually by separating the distribution in two compartments (either cytoplasm, or nucleus). Only clearly individualized particles were numerated. The minimum distance between isolated nanoparticles was taken at 40 nm, which is the maximal distance observed for nanoparticles attached to SWCNT. The number of isolated nanoparticles was then normalized to the surface area of the different compartments, in μm^2^, where they were identified. Data are presented as mean ± SEM.

### Synchotron-based microfluorescence experiments

For microfluorescence studies, murine cells were seeded at 250 000 cells/ml directly on thin window films used for micro X-ray fluorescence (XRF) studies [17] (Ultralene®, 4 μm thick, SPEX SamplePrep) and cultured for 24 hours as described above. These films were previously glued on specifically designed sample holders and sterilized by successive baths in 70° alcohol and antibiotics (penicillin/streptomycin). This biocompatible cell culture experimental system was put in 40 mm diameter cell culture Petri dishes and covered with cell suspension and cell culture medium. Twenty-four hours later, the cell culture medium was removed and the adherent cells monolayer was exposed for 3 to 24 hours to serum-free CNT suspensions (50 μg/ml), or to serum-free cell culture medium for unexposed samples, in presence or absence of 10 nM Concanamycin A.

At the end of exposure, cells were gently washed with 1× phosphate buffer saline (PBS, Gibco LifeTechnologies). Then, a quick wash with ultra-pure water (Sigma, Chromasolv® plus) was performed to remove salts coming from the culture medium. Immediately after, cells were further cryo-fixed and freeze-dried. Cryo-fixation was realized by rapid plunge of the cell culture system (cells on Ultralene film glued on sample holder) into an isopentane bath cooled with liquid nitrogen. Lyophilization was performed on a Christ Alpha 1–4 apparatus (ThermoFisher Scientific, Bioblock).

Synchrotron-based XRF microscopy experiments were performed on the ID21 beamline of the European Synchrotron Radiation Facility (ESRF, Grenoble, France). A fixed exit double crystal Si < 111 > monochromator (Kohzu) ensures a spectral resolution of 0.8 eV at 7.1 keV. Beam focalisation was assured by Kirkpatrick Baez (KB) mirrors allowing a beam size of 0.4*0.9 μm^2^. A silicon drift detector (Rontek) was used for fluorescence detection. An incident X-ray energy of 7.2 keV was chosen in order to excite the K-lines of elements of atomic number up to 26 (iron). All elements ranging from sodium (Z = 11) to iron (Z = 26) were detectable. Raster scans were performed across a chosen area of the sample preparation. From the analysis of the X-ray fluorescence spectrum for each pixel, a spatial image can be obtained for each element separately. Such an image represents a two-dimensional projection of the volumetric distribution of the elements. The vertical and horizontal pixel size was 0.5 μm each and data collection time for each pixel was about 0.5 s. Analysis of the fluorescence data has been performed in batch processing using the PyMca software [41].

### Acridine orange stain

Cells were seeded at 50 000 cells/well in 8-well cell culture chamber slide (Lab Tek, Nunc, ATGC Biotechnology, France), and cultured for 24 hours in serum condition before exposure. They were further exposed to 50 μg/ml SWCNT in presence or in absence of 10 nM Concanamycin A for 3 to 24 hours in serum-free conditions. At the end of the exposure, cells were washed twice in PBS and further incubated with a 5 μg/ml solution of Acridine Orange (Sigma-Aldrich, France) for 15 min at 37°C. They were then rinsed three times in PBS, mounted with a DAPI-containing anti-fading solution (Vectashield H1200, Vector, France) and immediately observed under a confocal microscope (Zeiss LSM 510 Meta coupled to Zeiss AxioObserver Z1 inversed microscope). Excitation was at 488 nm, and emission was at 610 nm (lysosomes in red) and at 530 nm (DNA/RNA in green).

### Cathepsin activity measurement

Cathepsin activity was measured using the cathepsin fluorogenic peptide substrate VII (R&D Systems, France), as previously described [19]. Cells were seeded at 80 000 cells/well on 96-well plates (TPP, ATGC Biotechnology, France) and cultured as described above. They were exposed to 10 or 50 μg/ml SWCNT for 3 hours or 24 hours in presence or in absence of 10 nM Concanamycin A. At the end of exposure, 50 μl of cell supernatant was harvested and incubated for 1 h at 37°C with 27 μM of Cathepsin fluorogenic substrate. The activity was then measured using a fluorimeter (Infinite M200 Pro, TECAN, France), with an excitation set-up at 380 nm and emission at 460 nm. Results are represented as box and whisker plots of at least 3 repeated experiments.

### Western-blot analysis

LAMP-1 and Cathepsin B protein expression were evaluated on cell lysates obtained after exposure of macrophages to SWCNT for 3 to 24 hours, in presence or in absence of 10 nM Concanamycin A. Samples were separated by SDS-PAGE and were transferred onto PVDF membranes. Blots were incubated with goat anti mouse LAMP-1 (AF965, R&D Systems, France), mouse anti mouse Cathepsin B (AF4320, R&D Systems), rabbit anti Phospho-p53 (Ser-15, CST 9284S, Cell Signaling), mouse anti γH2AX (phospho S139, Abcam, France), goat anti H-Ferritin (sc-14416, Santa Cruz, France), or rabbit anti HO-1 (3001, Biomol, France). ß-actin was used as internal standard. Quantification of the expression of each protein was obtained using the ratio over ß-Actin expression. Results are represented as box and whisker plots of at least 3 repeated experiments.

### Statistical analysis

Statistical evaluations were performed using GraphPad Prism software. As most of the data measured had a non-Gaussian distribution, the non-parametric Kruskall-Wallis test was used, with a Dunn’s Multiple Comparison post-hoc test when appropriated, or a Chi-square test. Significance was set at p < 0.05.

## Competing interests

The authors have no competing interests to declare.

## Authors’ contributions

CB, JC, JB, PL and SL designed the study. CB, EB, LA and SL performed the biological assays. CB, EP, JC, MH, MP, BF, MS, SL and PL performed the physicochemical experiments. CB, NB and CM performed the microscopy observations. SL drafted the manuscript; CB, EP, JC, JB and PL were the main additional participants to its further elaboration. All authors approved the final manuscript.

## Supplementary Material

Additional file 1: Figure S1 EELS spectrum of catalyst nanoparticle. Typical EELS spectrum taken on a CNT-attached iron-based nanoparticle, zoomed around the Fe2p edge. Click here for file

Additional file 2: Figure S2 HAADF-STEM images of unexposed and SWCNT-exposed cells. Panel **a**: representative HAADF-STEM images of unexposed macrophages. Panel **b**: representative HAADF-STEM images of SWCNT-exposed macrophages (higher magnification on cytoplasmic region). Panel **c**: representative HAADF-STEM images of the nucleus of SWCNT-exposed macrophages. Click here for file

Additional file 3: Figure S3 H-Ferritin and HO-1 expression. Panel **a**: typical western Blot image of H-Ferritin (23 kDa) and HO-1 (32 kDa) expression in macrophages exposed for 24 hours to SWCNT. ß-Actin is given as internal standard. Panel **b**: quantification of H-Ferritin and HO-1 expression in Western Blot, normalized to ß-Actin expression. Click here for file

## References

[B1] Ajayan PM Tour JM Materials science: nanotube composites Nature 2007 447 1066 1068 10.1038/4471066a 17597753

[B2] Lacerda L Bianco A Prato M Kostarelos K Carbon nanotubes as nanomedicines: from toxicology to pharmacology Adv Drug Deliv Rev 2006 58 1460 1470 10.1016/j.addr.2006.09.015 17113677

[B3] Mercer RR Hubbs AF Scabilloni JF Wang L Battelli LA Schwegler-Berry D Castranova V Porter DW Distribution and persistence of pleural penetrations by multi-walled carbon nanotubes Part Fibre Toxicol 2011 7 28 20920331 10.1186/1743-8977-7-28 PMC2958975

[B4] Murphy FA Schinwald A Poland CA Donaldson K The mechanism of pleural inflammation by long carbon nanotubes: interaction of long fibres with macrophages stimulates them to amplify pro-inflammatory responses in mesothelial cells Part Fibre Toxicol 2012 9 8 10.1186/1743-8977-9-8 22472194 PMC3352110

[B5] Tabet L Bussy C Setyan A Simon-Deckers A Rossi MJ Boczkowski J Lanone S Coating carbon nanotubes with a polystyrene-based polymer protects against pulmonary toxicity Part Fibre Toxicol 2011 8 3 10.1186/1743-8977-8-3 21255417 PMC3030506

[B6] Boczkowski J Lanone S Respiratory toxicities of nanomaterials - a focus on carbon nanotubes Adv Drug Deliv Rev 2012 64 1694 1699 10.1016/j.addr.2012.05.011 22641117

[B7] Johnston HJ Hutchison GR Christensen FM Peters S Hankin S Aschberger K Stone V A critical review of the biological mechanisms underlying the in vivo and in vitro toxicity of carbon nanotubes: the contribution of physico-chemical characteristics Nanotoxicology 2010 4 207 246 10.3109/17435390903569639 20795897

[B8] Donaldson K Poland CA Schins RP Possible genotoxic mechanisms of nanoparticles: criteria for improved test strategies Nanotoxicology 2010 4 414 420 10.3109/17435390.2010.482751 20925449

[B9] Porter DW Hubbs AF Mercer RR Wu N Wolfarth MG Sriram K Leonard S Battelli L Schwegler-Berry D Friend S Mouse pulmonary dose- and time course-responses induced by exposure to multi-walled carbon nanotubes Toxicology 2010 269 136 147 10.1016/j.tox.2009.10.017 19857541

[B10] Mercer RR Scabilloni J Wang L Kisin E Murray AR Schwegler-Berry D Shvedova AA Castranova V Alteration of deposition pattern and pulmonary response as a result of improved dispersion of aspirated single-walled carbon nanotubes in a mouse model Am J Physiol Lung Cell Mol Physiol 2008 294 L87 L97 18024722 10.1152/ajplung.00186.2007

[B11] Warheit DB Laurence BR Reed KL Roach DH Reynolds GA Webb TR Comparative pulmonary toxicity assessment of single-wall carbon nanotubes in rats Toxicol Sci 2004 77 117 125 14514968 10.1093/toxsci/kfg228

[B12] Muller J Huaux F Moreau N Misson P Heilier JF Delos M Arras M Fonseca A Nagy JB Lison D Respiratory toxicity of multi-wall carbon nanotubes Toxicol Appl Pharmacol 2005 207 221 231 10.1016/j.taap.2005.01.008 16129115

[B13] Shvedova AA Kisin ER Mercer R Murray AR Johnson VJ Potapovich AI Tyurina YY Gorelik O Arepalli S Schwegler-Berry D Unusual inflammatory and fibrogenic pulmonary responses to single-walled carbon nanotubes in mice Am J Physiol Lung Cell Mol Physiol 2005 289 L698 L708 10.1152/ajplung.00084.2005 15951334

[B14] Charron G Mazerat S Erdogan M Gloter A Filoramo A Cambedouzou J Launois P Rivière E Wernsdorfer W Bourgoin JP Mallah T Insights into the mechanism of the gas-phase purification of HiPco SWNTs through a comprehensive multi-technique study New J Chem 2009 33 1211 1223 10.1039/b900373h

[B15] Chorro M Kané G Alvarez L Cambedouzou J Paineau E Rossberg A Kociak M Aznar R Pascarelli S Launois P Bantignies JL 1D-confinement of polyiodides inside single-wall carbon nanotubes Carbon 2013 52 100 108

[B16] Heresanu V Castro C Cambedouzou J Pinault M Stephan O Reynaud C Mayne-L’hermite M Launois P Nature of the Catalyst Particles in CCVD Synthesis of Multiwalled Carbon Nanotubes Revealed by the Cooling Step Study J Phys Chem C 2008 112 7371 7378 10.1021/jp709825y

[B17] Bussy C Cambedouzou J Lanone S Leccia E Heresanu V Pinault M Mayne-L’hermite M Brun N Mory C Cotte M Carbon Nanotubes in Macrophages: Imaging and Chemical Analysis by X-ray Fluorescence Microscopy Nano Lett 2008 8 2659 2663 10.1021/nl800914m 18672943

[B18] Liu X Guo L Morris D Kane AB Hurt RH Targeted Removal of Bioavailable Metal as a Detoxification Strategy for Carbon Nanotubes Carbon N Y 2008 46 489 500 10.1016/j.carbon.2007.12.018 19255622 PMC2614279

[B19] Hamilton RF Jr Wu N Porter D Buford M Wolfarth M Holian A Particle length-dependent titanium dioxide nanomaterials’ toxicity and bioactivity Part Fibre Toxicol 2009 6 35 10.1186/1743-8977-6-35 20043844 PMC2806338

[B20] Garin J Diez R Kieffer S Dermine JF Duclos S Gagnon E Sadoul R Rondeau C Desjardins M The phagosome proteome: insight into phagosome functions J Cell Biol 2001 152 165 180 10.1083/jcb.152.1.165 11149929 PMC2193653

[B21] Huss M Wieczorek H Inhibitors of V-ATPases: old and new players J Exp Biol 2009 212 341 346 10.1242/jeb.024067 19151208

[B22] Zhu L Chang DW Dai L Hong Y DNA damage induced by multiwalled carbon nanotubes in mouse embryonic stem cells Nano Lett 2007 7 3592 3597 10.1021/nl071303v 18044946

[B23] Evstatiev R Gasche C Iron sensing and signalling Gut 2012 61 933 952 10.1136/gut.2010.214312 22016365

[B24] Allen BL Kichambare PD Gou P Vlasova II Kapralov AA Konduru N Kagan VE Star A Biodegradation of single-walled carbon nanotubes through enzymatic catalysis Nano Lett 2008 8 3899 3903 10.1021/nl802315h 18954125

[B25] Kagan VE Konduru NV Feng W Allen BL Conroy J Volkov Y Vlasova II Belikova NA Yanamala N Kapralov A Carbon nanotubes degraded by neutrophil myeloperoxidase induce less pulmonary inflammation Nat Nano 2010 5 354 359 10.1038/nnano.2010.44 PMC6714564 20364135

[B26] Liu X Hurt RH Kane AB Biodurability of Single-Walled Carbon Nanotubes Depends on Surface Functionalization Carbon N Y 2010 48 1961 1969 10.1016/j.carbon.2010.02.002 20352066 PMC2844903

[B27] Russier J Menard-Moyon C Venturelli E Gravel E Marcolongo G Meneghetti M Doris E Bianco A Oxidative biodegradation of single- and multi-walled carbon nanotubes Nanoscale 2011 3 893 896 10.1039/c0nr00779j 21116547

[B28] Kotchey GP Hasan SA Kapralov AA Ha SH Kim K Shvedova AA Kagan VE Star A A Natural Vanishing Act: The Enzyme-Catalyzed Degradation of Carbon Nanomaterials Acc Chem Res 2012 45 1770 1781 10.1021/ar300106h 22824066 PMC3473158

[B29] Andon FT Kapralov AA Yanamala N Feng W Baygan A Chambers BJ Hultenby K Ye F Toprak MS Brandner BD Biodegradation of Single-Walled Carbon Nanotubes by Eosinophil Peroxidase Small 2013 10.1002/smll.201202508 PMC4039041 23447468

[B30] Elgrabli D Floriani M Abella-Gallart S Meunier L Gamez C Delalain P Rogerieux F Boczkowski J Lacroix G Biodistribution and clearance of instilled carbon nanotubes in rat lung Part Fibre Toxicol 2008 5 20 10.1186/1743-8977-5-20 19068117 PMC2645433

[B31] Porter AE Muller K Skepper J Midgley P Welland M Uptake of C60 by human monocyte macrophages, its localization and implications for toxicity: studied by high resolution electron microscopy and electron tomography Acta Biomater 2006 2 409 419 10.1016/j.actbio.2006.02.006 16765881

[B32] Kang B Mackey MA El-Sayed MA Nuclear targeting of gold nanoparticles in cancer cells induces DNA damage, causing cytokinesis arrest and apoptosis J Am Chem Soc 2010 132 1517 1519 10.1021/ja9102698 20085324

[B33] Folkmann JK Risom L Jacobsen NR Wallin H Loft S Moller P Oxidatively damaged DNA in rats exposed by oral gavage to C60 fullerenes and single-walled carbon nanotubes Environ Health Perspect 2009 117 703 708 19479010 10.1289/ehp.11922 PMC2685830

[B34] Jacobsen NR Moller P Jensen KA Vogel U Ladefoged O Loft S Wallin H Lung inflammation and genotoxicity following pulmonary exposure to nanoparticles in ApoE−/− mice Part Fibre Toxicol 2009 6 2 10.1186/1743-8977-6-2 19138394 PMC2636756

[B35] Jacobsen NR Pojana G White P Moller P Cohn CA Korsholm KS Vogel U Marcomini A Loft S Wallin H Genotoxicity, cytotoxicity, and reactive oxygen species induced by single-walled carbon nanotubes and C(60) fullerenes in the FE1-Mutatrade markMouse lung epithelial cells Environ Mol Mutagen 2008 49 476 487 10.1002/em.20406 18618583

[B36] Kisin ER Murray AR Keane MJ Shi XC Schwegler-Berry D Gorelik O Arepalli S Castranova V Wallace WE Kagan VE Shvedova AA Single-walled carbon nanotubes: geno- and cytotoxic effects in lung fibroblast V79 cells J Toxicol Environ Health A 2007 70 2071 2079 10.1080/15287390701601251 18049996

[B37] Migliore L Saracino D Bonelli A Colognato R D’Errico MR Magrini A Bergamaschi A Bergamaschi E Carbon nanotubes induce oxidative DNA damage in RAW 264.7 cells Environ Mol Mutagen 2010 51 294 303 20091701 10.1002/em.20545

[B38] Beck-Speier I Kreyling WG Maier KL Dayal N Schladweiler MC Mayer P Semmler-Behnke M Kodavanti UP Soluble iron modulates iron oxide particle-induced inflammatory responses via prostaglandin E(2)synthesis: In vitro and in vivo studies Part Fibre Toxicol 2009 6 34 10.1186/1743-8977-6-34 20028532 PMC2806337

[B39] Kagan VE Tyurina YY Tyurin VA Konduru NV Potapovich AI Osipov AN Kisin ER Schwegler-Berry D Mercer R Castranova V Shvedova AA Direct and indirect effects of single walled carbon nanotubes on RAW 264.7 macrophages: Role of iron Toxicol Lett 2006 165 88 100 10.1016/j.toxlet.2006.02.001 16527436

[B40] Murray A Kisin E Leonard S Young SH Kommineni C Kagan VE Castranova V Shvedova A Oxidative stress and inflammatory response in dermal toxicity of single-walled carbon nanotubes Toxicol 2009 257 161 171 10.1016/j.tox.2008.12.023 19150385

[B41] Solé V Papillon E Cotte M Walter P Susini J A multiplatform code for the analysis of energy-dispersive X-ray fluorescence spectra Spectrochimica Actac Part B 2007 62 63 68 10.1016/j.sab.2006.12.002

