# Supplementary figures and images for: Intracellular fate of carbon nanotubes inside murine macrophages: pH-dependent detachment of iron catalyst nanoparticles
Source: Part Fibre Toxicol. 2013 Jun 25;10:24. doi: 10.1186/1743-8977-10-24 (PMC3699388; doi:10.1186/1743-8977-10-24)

Supplementary figure 1

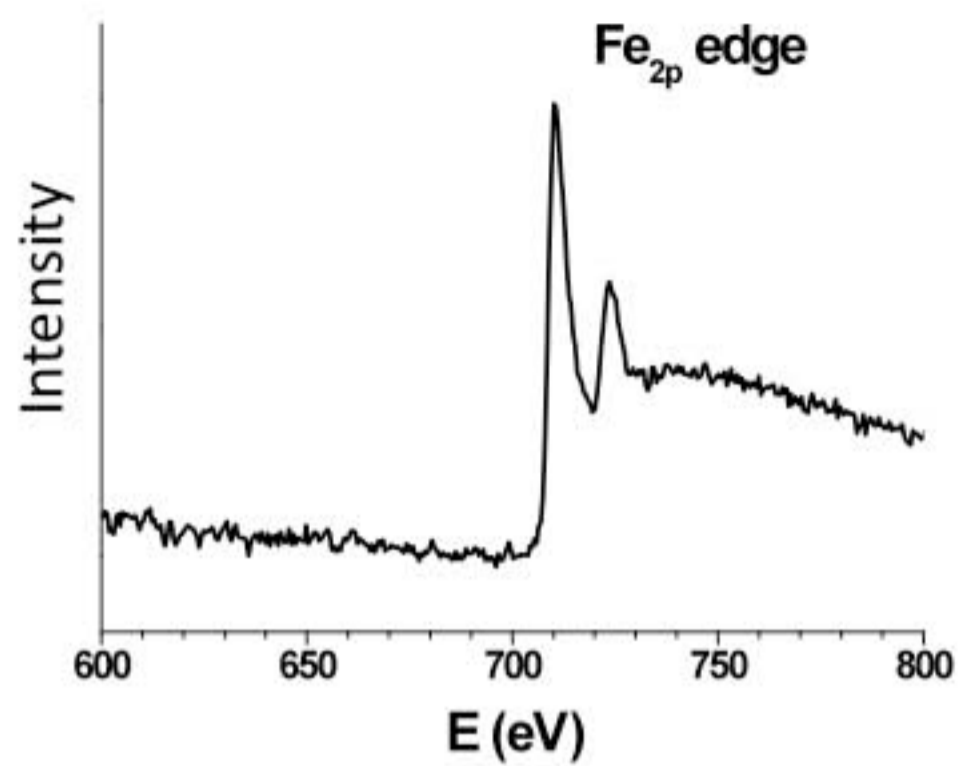

Supplement: Additional file 1: Figure S1 — EELS spectrum of catalyst nanoparticle. Typical EELS spectrum taken on a CNT-attached iron-based nanoparticle, zoomed around the Fe2p edge. [file 1743-8977-10-24-S1.pdf]

Supplementary figure 2

a

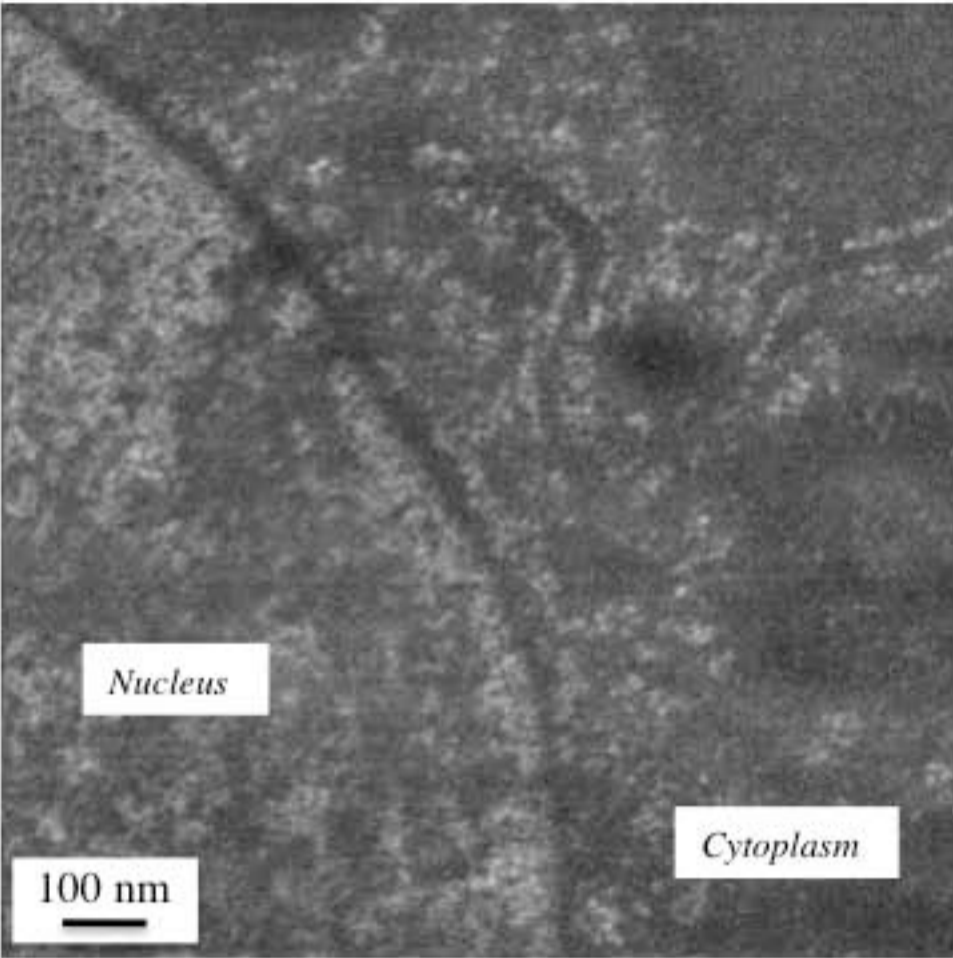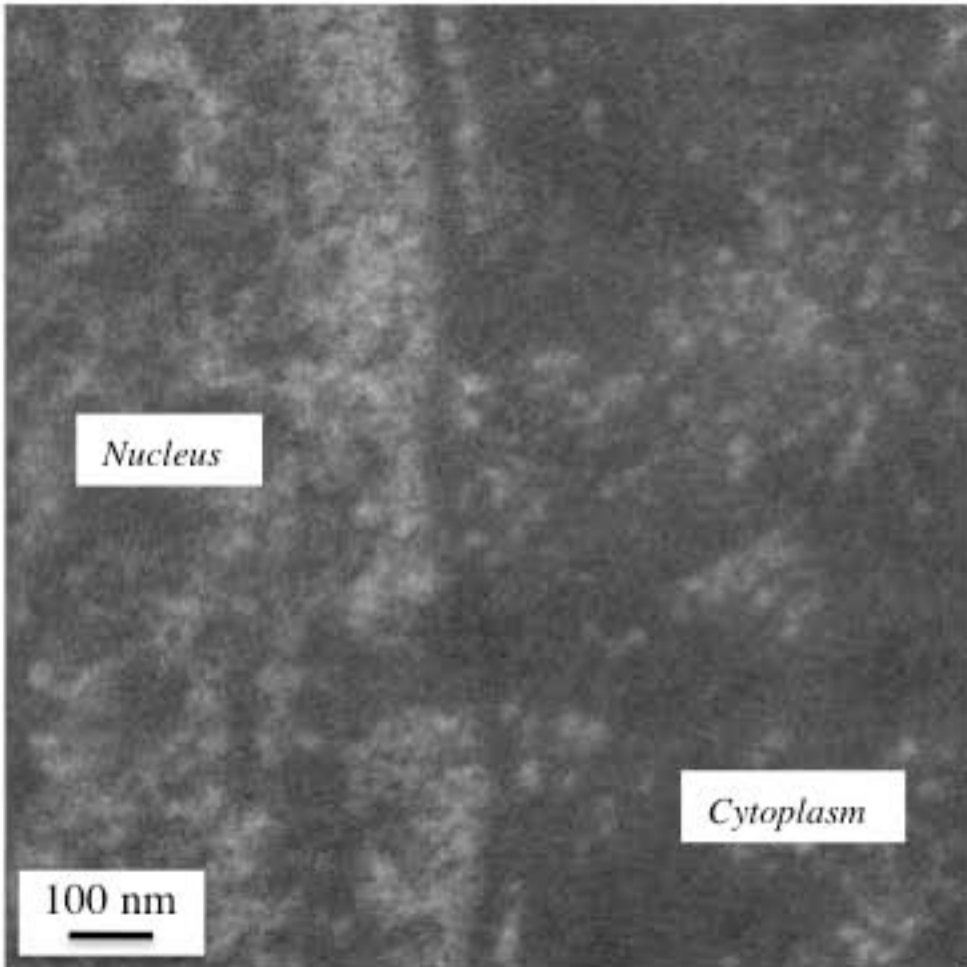

Supplementary figure 2

b

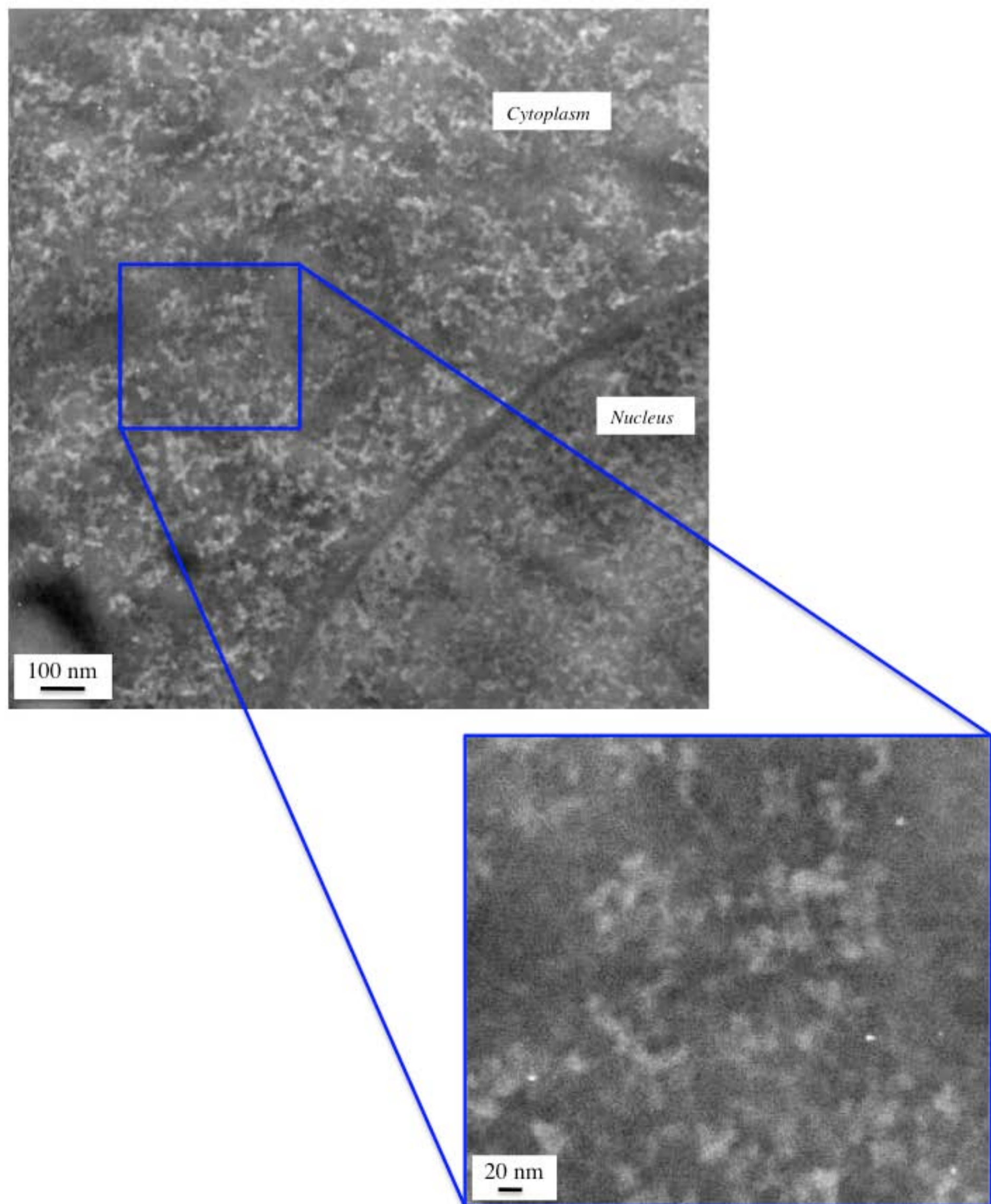

Supplementary figure 2

c

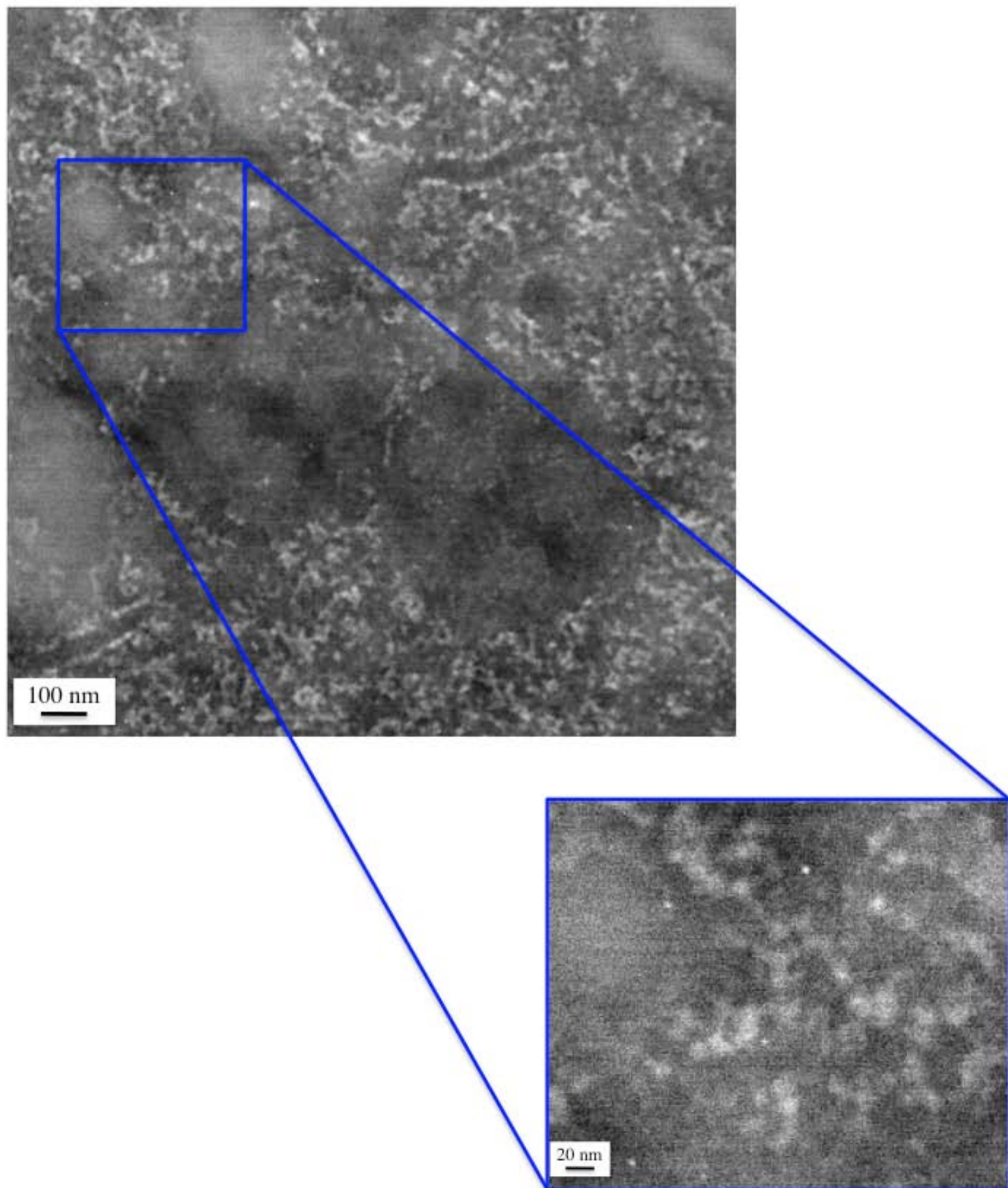

Supplement: Additional file 2: Figure S2 — HAADF-STEM images of unexposed and SWCNT-exposed cells. Panel a: representative HAADF-STEM images of unexposed macrophages. Panel b: representative HAADF-STEM images of SWCNT-exposed macrophages (higher magnification on cytoplasmic region). Panel c: representative HAADF-STEM images of the nucleus of SWCNT-exposed macrophages. [file 1743-8977-10-24-S2.pdf]

Supplementary figure 3

a

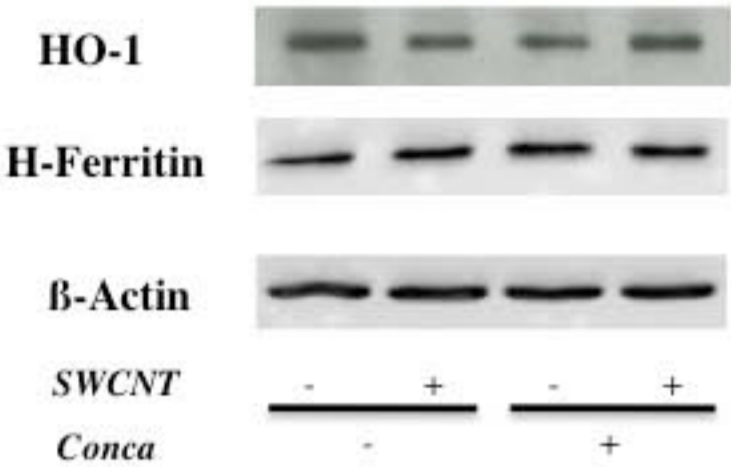

b

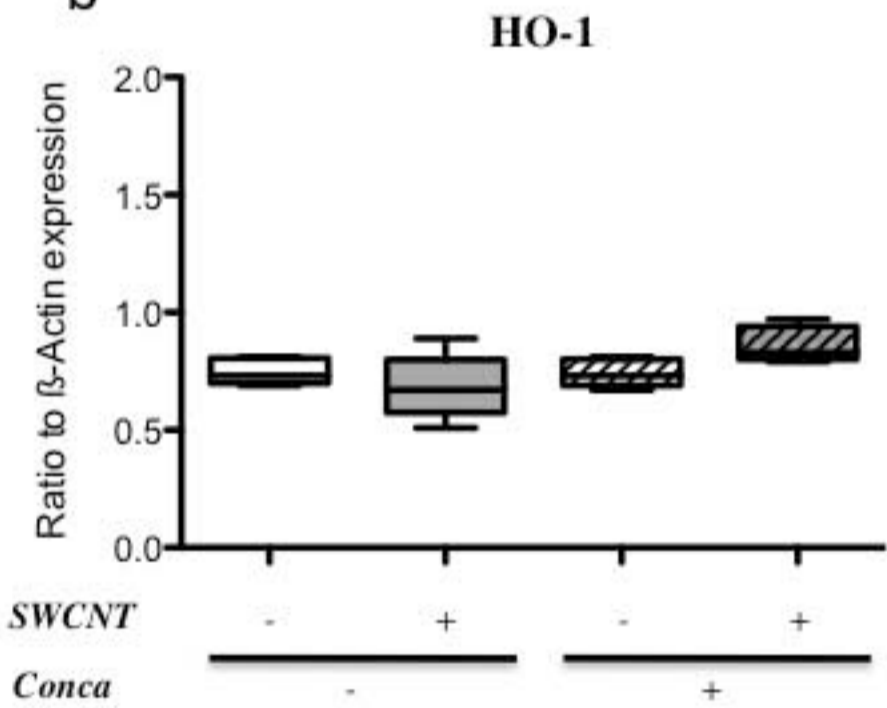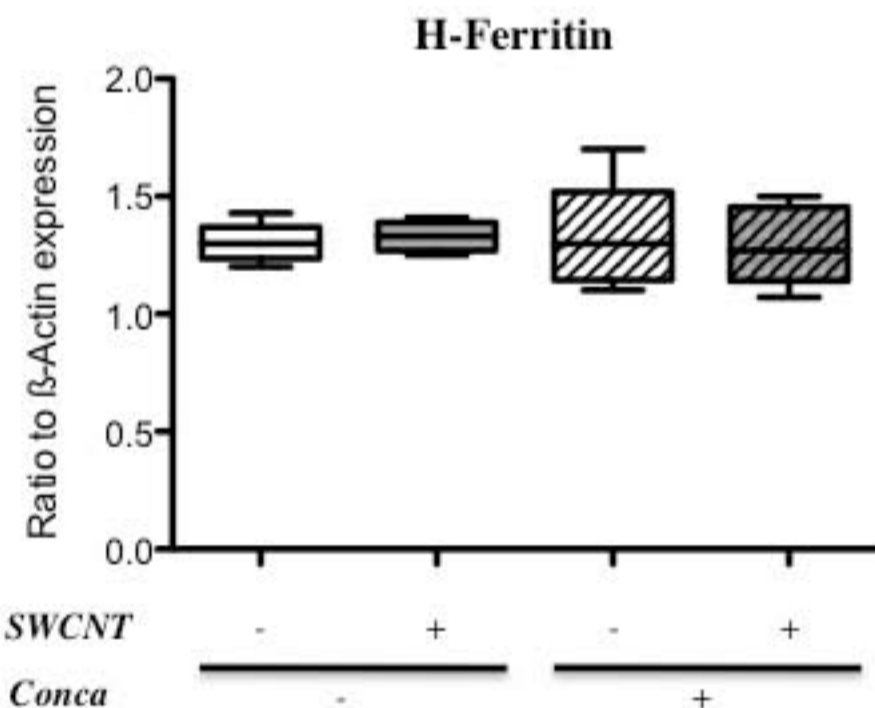

Supplement: Additional file 3: Figure S3 — H-Ferritin and HO-1 expression. Panel a: typical western Blot image of H-Ferritin (23 kDa) and HO-1 (32 kDa) expression in macrophages exposed for 24 hours to SWCNT. ß-Actin is given as internal standard. Panel b: quantification of H-Ferritin and HO-1 expression in Western Blot, normalized to ß-Actin expression. [file 1743-8977-10-24-S3.pdf]
